# Intraoperative Augmented Reality Visualization in Endoscopic Transsphenoidal Tumor Resection Using the Endoscopic Surgical Navigation Advanced Platform (EndoSNAP): A Technical Note and Retrospective Cohort Study

**DOI:** 10.7759/cureus.79714

**Published:** 2025-02-26

**Authors:** Lea B Tortolero, Sabastian A Hajtovic, Jose Gautreaux, Richard Lebowitz, Dimitris G Placantonakis

**Affiliations:** 1 Neurological Surgery, NYU Langone Health, New York, USA; 2 Neurosurgery, St. Barnabas Hospital Health System, Bronx, USA; 3 Neurosurgery, Northwell Health, Manhasset, USA; 4 Neurosurgery, NYU Grossman School of Medicine, New York, USA; 5 Otolaryngology, NYU Grossman School of Medicine, New York, USA

**Keywords:** augmented reality (ar), endoscopic transsphenoidal approach (etsa), image-guided neurosurgery, pituitary tumors, skull base tumors, surgical theater, virtual reality (vr)

## Abstract

The endoscopic transsphenoidal approach (ETSA) is a commonly used technique that allows for the minimally invasive removal of sellar and parasellar lesions. Augmented reality (AR) applications in ETSA are hypothesized to enhance intraoperative visualization by integrating a 3D-reconstructed model into the operative field. This study describes the workflow and surgical outcomes associated with the Endoscopic Surgical Navigation Advanced Platform (EndoSNAP, Surgical Theater, Cleveland, OH, USA), an AR platform designed for surgical planning and intraoperative navigation in ETSA for sellar and parasellar lesions. We analyzed a cohort of patients who underwent ETSA tumor resection using EndoSNAP. Preoperative MRI and CT scans were reconstructed and merged into a single 360° AR model using the Surgical Rehearsal Platform software. The model was then imported into EndoSNAP, which was integrated with the endoscope and neuronavigation system for real-time intraoperative use. Patient demographics, tumor characteristics, extent of resection (EOR), and endocrinologic and neurologic outcomes were recorded. Eighteen adult patients with newly diagnosed (83%) and recurrent (17%) tumors were included. Pathologies consisted of pituitary adenoma (72%), craniopharyngioma (11%), meningioma (11%), and chordoma (6%). Optic compression was present in 56% of patients, with preoperative visual deficits in 70% of them. Cavernous sinus invasion was observed in 17% of tumors. Preoperative hormonal excess and insufficiency were noted in 56% and 28% of cases, respectively. The mean preoperative tumor volume was 21.4 ± 17 cm³, which decreased to 0.4 ± 0.3 cm³ postoperatively. The mean EOR was 93.6 ± 3.6%. Postoperative complications included CSF leaks requiring surgical repair (17%), seizures related to pre-existing hemispheric trauma (6%), pulmonary embolism (6%), deep venous thrombosis (6%), and sinusitis (6%). These findings suggest that AR-enhanced visualization through EndoSNAP is a feasible and potentially beneficial adjunct in ETSA for sellar and parasellar tumor resection.

## Introduction

The endoscopic transsphenoidal approach (ETSA) is a minimally invasive technique for treating sellar and parasellar lesions [1]. First described over three decades ago [2], ETSA has facilitated a global shift from microsurgical to fully endoscopic approaches [3-5]. Accessing the sellar and parasellar regions through the sphenoid sinus provides a direct and panoramic view of critical structures, including the pituitary gland and stalk, infundibulum and hypothalamus, optic nerves and chiasm, internal carotid arteries (ICAs) and cavernous sinuses, as well as upper retroclival vessels and cranial nerves. The efficacy and safety of ETSA have been well established for various skull base pathologies, with studies reporting higher gross total resection rates, shorter postoperative hospital stays, and lower complication rates compared to traditional microsurgical approaches [6-11]. Notably, ETSA has been shown to outperform microscopic surgery for pituitary adenomas with parasellar extension [7], with reported mortality rates as low as 0.4% [12]. Additionally, this approach is viable in emergency scenarios, such as pituitary apoplexy [13].

Image-guided surgery (IGS) is widely employed in ETSA to enhance the localization of critical structures that may be obscured in the limited operative field [14]. IGS increases surgical precision by allowing real-time tracking of the endoscope relative to preoperative imaging. However, conventional IGS has inherent limitations. It relies on 2D preoperative radiologic images to navigate a 3D anatomical space, requiring surgeons to mentally reconstruct a 3D model to understand the pathology’s relationship with surrounding structures. This mental reconstruction introduces potential errors, particularly in the complex operating room environment. Moreover, the need to reference an external monitor forces surgeons to shift focus away from the surgical field. The accuracy of this 2D-to-3D interpretation can influence complication risks, which vary based on lesion type, size, proximity to critical structures, and surgeon experience. These risks are further heightened in re-operations due to anatomical distortions and adhesions [15].

Augmented reality (AR) has recently emerged as a tool for improving intraoperative visualization and guidance in surgery. AR overlays digital information onto the real environment, allowing simultaneous interaction with virtual and physical stimuli. The AR display can be presented in 2D or 3D on an external monitor or directly within the surgical field. Several AR applications have been explored for endoscopic skull base and sinus surgery [16-19]. The first reported use of AR in endoscopic surgery dates back to 2002, when 3D wireframe models of tumors and key anatomical structures were superimposed on real-time endoscopic images to aid transsphenoidal pituitary tumor resection [18]. More recent approaches utilize segmentation software to overlay virtual anatomical information onto the endoscopic view [16,17,19]. One study demonstrated an extended AR view in which 3D anatomical models were superimposed onto the endoscopic skull base visualization, achieving a mean spatial accuracy error of approximately 1 mm [20]. Despite these advancements, AR applications in endoscopic surgery have not been widely adopted.

The Endoscopic Surgical Navigation Advanced Platform (EndoSNAP, Surgical Theater, Cleveland, OH, USA) integrates AR capabilities into endoscopic procedures within the operating room [21]. Utilizing a patient’s preoperative imaging, EndoSNAP generates a 3D reconstruction to assist in both surgical planning and intraoperative navigation. Before surgery, the patient’s 360° model is imported into EndoSNAP, where it is used intraoperatively to enhance visualization beyond the limitations of the endoscope. This enables the surgeon to see around, behind, and through critical structures. EndoSNAP synchronizes with the endoscope and image guidance system to provide real-time spatial awareness, enhancing both situational awareness and surgical precision. By capturing and enhancing the endoscopic video feed, patient-specific plans can be viewed in on-screen AR and immersive virtual reality (VR).

In this study, we present our experience with the EndoSNAP AR platform for surgical planning and intraoperative navigation in ETSA. We outline its integration into our workflow for the resection of various pathologies, including pituitary adenoma, craniopharyngioma, meningioma, and chordoma. Additionally, we evaluate surgical outcomes and complications associated with these procedures.

## Materials and methods

Data collection

We prospectively compiled a database of 18 consecutive adult patients who underwent ETSA tumor resection using EndoSNAP (Surgical Theater) integrated with the Brainlab Curve navigation system (Brainlab, Munich, Germany). Surgeries were performed by DGP and RL between May 2018 and July 2020. Retrospective chart review was approved by the IRB (approval number 11-01733). Collected clinical data included patient demographics, neurologic and endocrinologic status, tumor pathology and location, preoperative and postoperative tumor volume, operative characteristics, and complications. Volumetric analysis of contrast-enhancing tumors before and after surgery was conducted using the Brainlab iPlan platform.

360° case rendering and preoperative planning with Surgical Rehearsal Platform (SRP)

**Figure 1:**
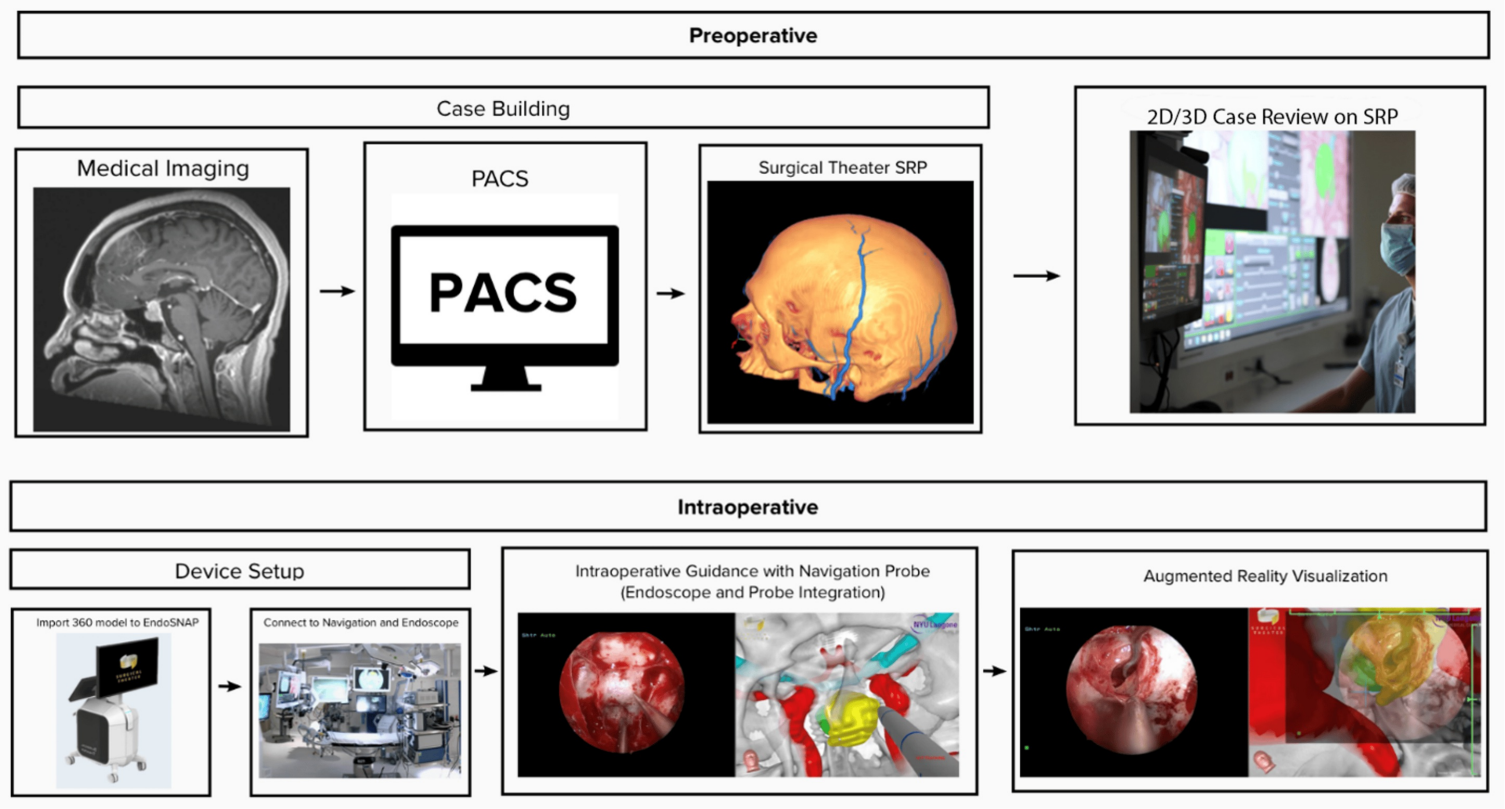
Workflow diagram illustrating the process from preoperative case construction to intraoperative implementation of the 360° model PACS, picture archiving and communication system; SRP, Surgical Rehearsal Platform

The workflow from preoperative imaging to intraoperative AR visualization is summarized in Figure 1. Each high-resolution 360° model was generated using patient-specific CT and MRI scans, which were reconstructed and merged into a single 360° AR model using the SRP software (Surgical Theater). On average, the creation of each 360° AR model took approximately 20 minutes.

Each scan contained voxel-based intensity variations, enabling a clinical specialist to semi-automatically segment structural elements from the original imaging. The first set of structures, including the skull and sinuses, was derived from the CT scan. The sinuses were typically extracted by adjusting the CT scan’s intensity range to display only the skin and air-filled spaces. The second set of structures, consisting of the brain and pathology, was segmented from the MRI scan. These elements were extracted as individual layers, allowing for independent manipulation of each structure.

Opacity settings for each structure were adjusted within the SRP according to the surgeon’s preferences. The pituitary gland, optic nerves and chiasm, surgical lesion, and ICAs were manually segmented from the MRI scan using the freehand drawing tool. The final 360° model was reviewed and approved by the surgeon for preoperative planning and navigation.

For each case, the surgeon evaluated the 360° model within the SRP to refine the optimal surgical approach. This review could be conducted either on a 2D platform or in 3D using VR technology with specialized goggles, providing an immersive and detailed perspective of the surgical anatomy.

AR visualization with EndoSNAP

**Figure 2:**
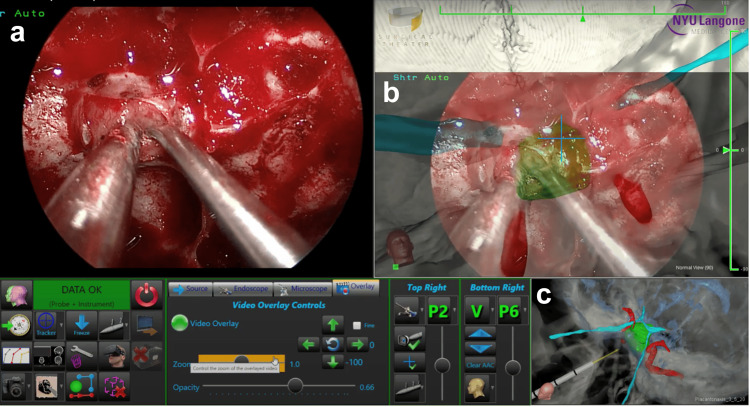
EndoSNAP screen layout displaying the following in each of the IGs (a) Live video feed from the endoscope. (b) Corresponding Surgical Theater 360° model with the endoscope video overlaid at 66% opacity for improved visualization. (c) Surgical Theater 360° model displaying the endoscope tool within the surgical field to enhance depth perception. EndoSNAP, Endoscopic Surgical Navigation Advanced Platform; IG, image generator

The 360° model was imported from the SRP into EndoSNAP, which was connected to both the endoscope and the Brainlab Curve navigation system for intraoperative use. After patient registration with the navigation system, EndoSNAP tracked the position and orientation of the pointer during key portions of the surgery. Integration with Brainlab navigation required attaching a tracking device with three reference spheres to the endoscope. The system captured the live endoscopic video feed and displayed it alongside the 360° model, with the endoscopic view appearing on the left image generator (IG) of the monitor and the corresponding 360° model on the two right IGs (Figure 2). Since EndoSNAP and the navigation system were synchronized, the 360° model adjusted dynamically relative to the endoscope, providing the surgeon with enhanced situational awareness.

Several advanced features were available to the surgeon during the procedure. One feature allowed the live endoscopic video feed to be overlaid onto the 360° model, with adjustable opacity to improve visualization of critical structures in relation to the endoscopic perspective. Another feature enabled the surgeon to change the tracked tool from the endoscope to an alternative instrument. For instance, when using the navigation probe, the surgeon could select “Probe” as the “Tracker for Camera Attachment,” shifting the displayed view to correspond with the probe’s tip. In Figure 2, only the top right IG would change based on the tracked tool, while the bottom right IG remained fixed, displaying the tool’s interaction with the model. For improved depth perception, the surgeon sometimes opted to keep a sagittal view in the fixed IG.

**Figure 3:**
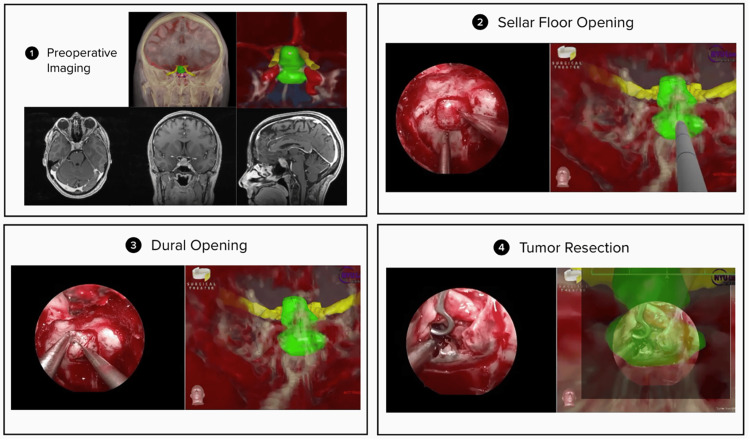
Operative stages of transsphenoidal pituitary adenoma resection utilizing EndoSNAP EndoSNAP, Endoscopic Surgical Navigation Advanced Platform

In all included cases, one or more of these advanced features were utilized. Figure 3 demonstrates the application of the patient-specific 360° model and EndoSNAP during the resection of a pituitary adenoma.

Statistical analysis

Statistical analysis was conducted using Prism 10 (GraphPad, Boston, MA, USA). Data normality was assessed with the D’Agostino-Pearson test. Since preoperative and postoperative tumor volumes, as well as extent of resection (EOR), did not follow a normal distribution, their descriptive statistics are reported as both the median (25th quartile and 75th quartile) and mean ± SEM. For all other variables, descriptive statistics are presented as mean ± SEM. A significance threshold of p < 0.05 was applied.

## Results

A total of 18 cases were included (Table 1). The mean age ± SEM was 48.8 ± 4.1 years, and 11 patients (61%) were female. Pituitary adenoma was the most common diagnosis, occurring in 13 patients (72%), including four (22%) prolactinomas and three (17%) GH-secreting adenomas in individuals with acromegaly. A detailed breakdown of tumor types is provided in Table 1. Additionally, two patients (11%) had craniopharyngioma, two (11%) had meningioma, and one (6%) had a chordoma. Tumors were newly diagnosed in 15 patients (83%) and recurrent in three patients (17%), all of whom had nonfunctioning pituitary macroadenomas

**Table 1:** Patient demographics, clinical features, and tumor characteristics CN, cranial nerve; DI, diabetes insipidus; EEA, endoscopic endonasal approach; F, female; GH, growth hormone; IGF1, insulin-like growth factor 1; Lt, left; M, male; mo., months; pit., pituitary; PRL, prolactin The asterisk (*) denotes a recurrent tumor.

Patient no.	Age	Sex	Pathology	Optic apparatus compression	Cavernous sinus invasion	Preop CN deficit	Preop hormone excess	Preop hormone insufficiency	Postop hormone status	Postop DI
1	69	M	Craniopharyngioma	Yes	No	Bitemporal visual deficit, Lt CN IV	None	Low testosterone	Pan-hypopituitarism	Permanent
2	20	F	GH-secreting pit. macroadenoma	No	No	None	GH, IGF1 (826)	Irregular menses	Reduced but abnormal GH, IGF1 (511 at five months)	No
3	38	F	Hemorrhagic pit. macroprolactinoma	Yes	No	None	PRL (169)	Amenorrhea	Normal menses returned	Transient
4	61	M	Planum sphenoidale meningioma	No	No	None	None	None	Normal	No
5	52	F	GH-secreting pit. microadenoma	No	No	None	IGF1 (219)	None	Persistent IGF1 elevation	No
6	79	M	Chordoma	No	No	None	None	None	Normal	No
7	50	F	Nonfunctioning pit. macroadenoma*	Yes	No	None	PRL (38)	None	Normal	Transient
8	56	M	Tuberculum sellae meningioma	No	No	None	None	None	Normal	No
9	27	F	Microprolactinoma	No	No	None	PRL (79)	Amenorrhea	Normal	No
10	38	F	Nonfunctioning pit. macroadenoma	Yes	No	Bitemporal visual deficit	PRL (67)	Amenorrhea	Normal	No
11	30	F	Nonfunctioning pit. macroadenoma	Yes	Unilateral	None	PRL (74)	Amenorrhea, hypothyroidism	GH/IGF1 deficiency, hypothyroidism	No
12	42	M	Craniopharyngioma	Yes	No	Bitemporal visual deficit	None	None	Pan-hypopituitarism	Permanent
13	77	M	Nonfunctioning pit. macroadenoma*	Yes	Bilateral	Binocular visual deficit, Lt CN III	None	Low testosterone	Low testosterone	No
14	68	M	Nonfunctioning pit. macroadenoma*	Yes	No	Lt temporal visual deficit	None	Low cortisol, hypothyroidism	Low cortisol, hypothyroidism	Transient
15	29	F	Microprolactinoma	No	No	None	PRL (150)	Amenorrhea, galactorrhea	Normal	No
16	55	F	Nonfunctioning pit. macroadenoma	Yes	No	Bitemporal and Lt nasal visual deficits	None	Low cortisol	Low cortisol, recovered	No
17	46	F	Cystic, hemorrhagic macroprolactinoma	No	No	None	PRL (123)	Amenorrhea	Regular menstrual cycle	No
18	41	F	Giant GH-secreting pit. adenoma	Yes	Bilateral	Lt. homonymous hemianopsia	GH (5.8) IGF1 (614)	None	Pan-hypopituitarism, IGF1 normal (204 at six months)	Permanent

Compression of the optic apparatus was present in 10 patients (56%), with preoperative visual deficits observed in seven of them (70%). Additionally, two patients (11%) exhibited other cranial nerve deficits, including one with a trochlear deficit and another with an oculomotor deficit. Cavernous sinus invasion was identified in three patients (17%), with one case involving unilateral invasion and two cases involving bilateral invasion.

Preoperative hormone excess was noted in 10 patients (56%), primarily involving elevated prolactin and insulin-like growth factor 1. Hormone insufficiency was observed in five patients (28%), including deficiencies in testosterone, thyroid hormone, and cortisol. Following tumor resection, six patients (33%) experienced complete resolution of their preoperative hormonal imbalance. However, three patients (17%) developed postoperative panhypopituitarism. Postoperative diabetes insipidus (DI) occurred in six patients (33%), with three cases being permanent. Among those with permanent DI, two had craniopharyngiomas, and one had a giant pituitary adenoma.

**Figure 4:**
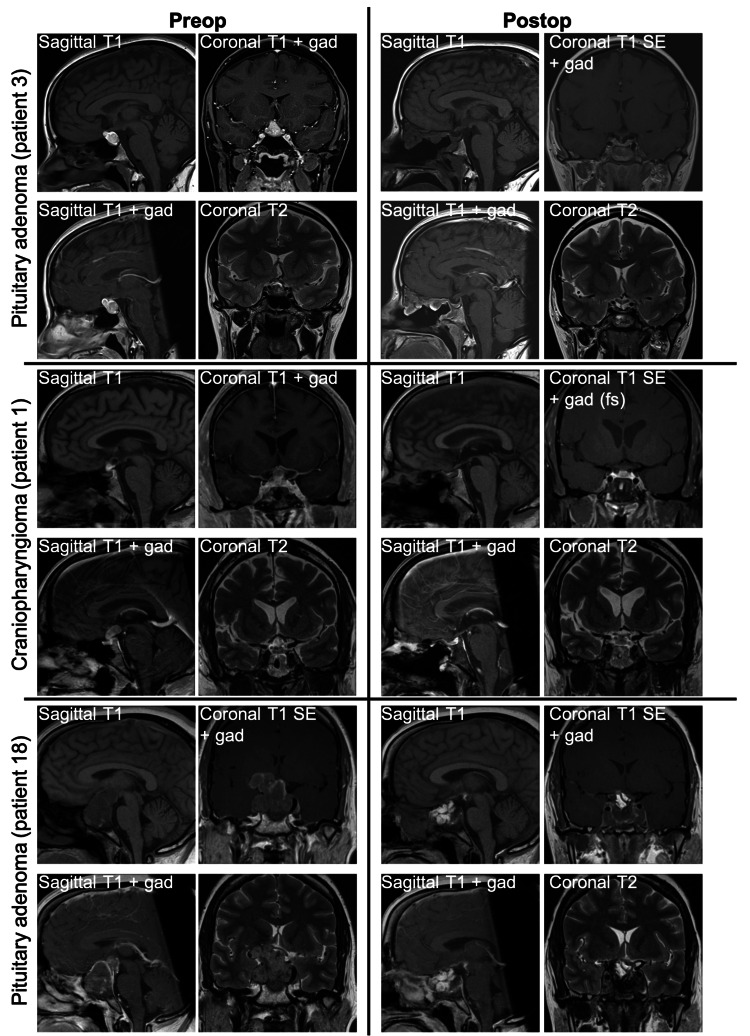
Examples of surgical outcomes achieved using EndoSNAP Preoperative and postoperative MRI images are displayed. The T1 hyperintensities observed in the surgical cavity of patient 18 on postoperative images indicate the presence of a fat graft. EndoSNAP, Endoscopic Surgical Navigation Advanced Platform; fs, fat suppression; gad, gadolinium; SE, spin echo

Mean preoperative and postoperative tumor volumes were 21.4 ± 17 cm³ and 0.4 ± 0.3 cm³, respectively, yielding a mean EOR of 93.6 ± 3.6%. Median preoperative and postoperative tumor volumes were 2.4 cm³ (0.8, 6) and 0 cm³ (0, 0.1), respectively, with a median EOR of 100% (96.6, 100). Complete tumor resection (EOR of 100%) was achieved in 12 of 18 patients (67%) (Table 2), while only three patients (17%) had an EOR below 95%. Examples of resection outcomes are illustrated in Figure 4, including a notable case involving near-total resection of a giant GH-secreting pituitary adenoma with a large suprasellar extension reaching the right basal ganglia.

**Table 2:** Operative characteristics, complications, and volumetric resection estimates DVT, deep venous thrombosis; EOR, extent of resection; PE, pulmonary embolus

Patient no.	Lumbar drain placement	Intraoperative CSF leak	Fat graft	Other postop complications	Preop tumor volume (cm³)	Postop tumor volume (cm³)	EOR (n%)
1	Yes	Yes	Yes	None	1.05	0	100%
2	No	Yes	No	None	1.05	0.03	97.14%
3	No	Yes	No	None	1.88	0	100%
4	Yes	Yes	Yes	CSF leak repair, DVT	3.09	0	100%
5	No	No	No	None	0.34	0.16	52.94%
6	Yes	Yes	Yes	None	7.984	0	100%
7	No	No	No	None	5.6	0	100%
8	Yes	Yes	No	None	0.77	0	100%
9	No	No	No	None	0.05	0	100%
10	No	Yes	No	None	2.76	0	100%
11	No	Yes	No	None	4.23	0.18	95.01%
12	Yes	Yes	Yes	CSF leak repair	3.82	0.02	99.33%
13	No	Yes	No	PE, sinusitis	7.19	3.55	50.63%
14	Yes	Yes	No	None	5.409	0	100%
15	No	No	No	None	0.16	0	100%
16	No	Yes	No	Seizure	2.01	0	100%
17	No	No	No	None	0.81	0	100%
18	Yes	Yes	Yes	CSF leak repair, bacterial meningitis	30.687	3.188	89.61%

Intraoperative CSF leaks occurred in 13 of 18 patients (72%), with fat graft placement performed in five of these cases (38%). Lumbar drains were used in seven patients (39%). Postoperative complications included CSF leaks requiring surgical repair in three patients (17%). One of these patients developed bacterial meningitis secondary to CSF rhinorrhea but fully recovered. Additionally, one patient with a preexisting seizure disorder due to prior hemispheric trauma experienced a seizure in the acute postoperative period unrelated to the surgery. Postoperative medical complications included one case of deep venous thrombosis and another case involving both pulmonary embolism and sinusitis.

## Discussion

Neurosurgery is a constantly evolving specialty driven by technological advancements that enhance patient safety and surgical outcomes. While the ETSA offers numerous benefits, it also carries risks of severe complications, such as ICA injury [22]. In recent years, AR applications have been explored to improve preoperative planning and intraoperative visualization, addressing the limitations of traditional image guidance in ETSA and other cranial procedures [16,17,19,21,23]. Despite these advancements, the global adoption of AR technology in neurosurgery remains in its early stages.

One inherent challenge of the ETSA is the constrained line of sight provided by the endoscope, which can impair surgical orientation and contribute to mental fatigue, potentially affecting surgical outcomes. To mitigate this limitation, Bong et al. developed a phantom model incorporating an extended AR navigation system [20]. This system enabled real-time overlay of a 3D-reconstructed image from preoperative imaging onto the endoscopic view, expanding the surgical field beyond the boundaries of the endoscope. The authors concluded that this approach enhanced both safety and precision in ETSA procedures [20]. Similarly, our EndoSNAP 3D model could be visualized on an external monitor or integrated into the endoscopic live view. The model’s opacity could be adjusted to the surgeon’s preference, improving anatomical understanding and spatial orientation during skull base tumor resections, a benefit also noted by other researchers [24].

In this study, we describe our technical experience and workflow for resecting sellar and parasellar lesions using EndoSNAP. Our findings suggest that this technology facilitates tumor resection by providing a 3D reconstruction from preoperative imaging, with annotations of critical structures such as the optic chiasm and ICAs. The ability to match the endoscopic video feed with the 3D model enhances spatial and situational awareness as the resection progresses. In a separate study, Zeiger et al. evaluated the mixed-reality application of EndoSNAP across a variety of endoscopic skull base surgeries. Surgeons reported that EndoSNAP was highly versatile and particularly useful for identifying critical structures and guiding the extent of bony dissection, especially in cases with altered anatomy due to pathology [21].

Research has demonstrated that AR integration in ETSA reduces the cognitive load on surgeons [17,19]. One possible explanation is that displaying a patient-specific 3D image directly onto the surgical field eliminates the need for mental reconstruction and minimizes the need to shift focus away from the operative site. This, in turn, may reduce cognitive effort and decrease the risk of errors. In a preclinical trial, Dixon et al. developed a live IGS system for endoscopic skull base approaches in cadaveric specimens. Surgeons reported significantly lower mental demand, effort, and frustration when using this system compared to traditional IGS [19]. Additionally, the incorporation of anti-target sounds - alerts for structures to be avoided - further enhanced surgical safety and confidence, enabling more aggressive resections [19]. Zeiger et al. similarly noted that surgeons felt more confident in tissue ablation when guided by AR, potentially improving intraoperative decision-making, EOR, and overall surgical efficiency [21]. AR technology has also been successfully applied to other neurosurgical procedures, including awake craniotomies [23].

Beyond intraoperative guidance, AR and VR applications play a significant role in medical education and resident training [25]. Neurosurgical simulation programs have been tested worldwide, offering trainees an innovative platform to practice new skills in a risk-free environment [26,27]. These simulations enable repeated practice and continuous review of complex anatomical relationships, ultimately aiming to improve surgical competency and patient outcomes. For example, the National Research Council of Canada developed NeuroTouch, a VR simulator for neurosurgical training, including modules for ETSA [26]. Preliminary data suggest that neurosurgery residents outperform medical students in certain surgical tasks using this system, highlighting its potential as an assessment tool [28]. Another study found significant competency differences between junior and senior residents, reinforcing the system’s utility for both skill evaluation and ongoing education [27]. These findings underscore the importance of incorporating AR and VR into neurosurgical training curricula to refine technical skills and enhance overall proficiency.

Despite these promising applications, augmented and mixed reality systems also present challenges [29]. First, in the context of surgical simulation training, formal integration into residency programs requires extensive validation [28], necessitating financial investment from institutions. Second, the cost of implementing AR technology may not provide immediate cost savings or revenue generation, which can hinder widespread adoption. Third, AR systems require considerable space in the operating room, posing logistical challenges for some institutions. Lastly, concerns have been raised about potential delays in projecting augmented images, which could impact surgical workflow [30].

This study also has several limitations. The small sample size and lack of a control group prevent definitive conclusions about the clinical and surgical benefits of EndoSNAP-guided resections compared to traditional IGS. Additionally, EndoSNAP was not used throughout the entirety of each procedure, making it difficult to isolate its impact from the surgeon’s expertise. Furthermore, selection bias may have influenced reported outcomes. Future research should focus on larger prospective studies with appropriate control groups and standardized intraoperative use of EndoSNAP to better assess its impact on surgical safety and efficacy.

Moving forward, research should explore the effects of this technology on surgeon cognitive load, confidence, and accessibility across different institutions and regions. Further studies should also evaluate its role in improving tumor resection rates and preserving critical structures, particularly in complex or highly invasive tumors. By addressing these questions, we can better understand the full potential of AR in advancing neurosurgical care.

## Conclusions

AR is emerging as a transformative tool in medicine, significantly enhancing surgical planning and execution in neurosurgery. In this study, we present our technical experience and workflow using AR in ETSAs for the resection of sellar and parasellar lesions. Our findings demonstrate that integrating AR into the operating room can facilitate skull base tumor resections, particularly in complex cases. Additionally, AR offers a streamlined, user-friendly system that can be adjusted to the surgeon’s preference during surgery. Numerous studies have shown that AR reduces cognitive load and frustration among surgeons. Moreover, by highlighting critical structures, AR may increase surgical confidence, allowing for more extensive resections and potentially improving patient outcomes. While AR is not a standalone solution, it is a valuable addition to the neurosurgical toolkit, complementing traditional techniques and enhancing tumor resection strategies.
